# The role and mechanisms of AMPK in neurovascular unit injury in Parkinson’s disease

**DOI:** 10.3389/fnagi.2026.1790159

**Published:** 2026-05-18

**Authors:** Bingqian Liu, Nan Li, Gaofang Fan, Zhifu Guo, Ke Wang, Lingyao Kong

**Affiliations:** Department of Neurology, The Second Affiliated Hospital of Zhengzhou University, Zhengzhou, Henan, China

**Keywords:** AMPK, blood-brain barrier, Parkinson’s disease, phosphoproteomics, α-synuclein

## Abstract

Parkinson’s disease (PD) is the second most common neurodegenerative disorder, characterized by dopaminergic neuron loss and α-synuclein aggregation. Increasing evidence indicates that blood-brain barrier (BBB) dysfunction in the neurovascular unit (NVU) is an early pathological event driving PD progression, rather than a secondary consequence. AMP-activated protein kinase (AMPK), a key cellular metabolic sensor, is activated by oxidative stress and endoplasmic reticulum stress induced by α-synuclein aggregates, and participates in regulating endothelial barrier function. This review systematically summarizes the pathological evidence of early BBB injury in PD, elaborates on the dual role of AMPK in BBB regulation, and highlights the value of phosphoproteomics in deciphering AMPK’s downstream signaling networks. We propose the “α-synuclein-AMPK-phosphorylation network-BBB dysfunction” axis, discuss pharmacological clues from traditional Chinese medicine, and outline future research directions. This review provides a novel integrated perspective for understanding PD neurovascular pathology and lays a theoretical foundation for identifying early intervention targets by targeting AMPK-regulated NVU protection.

## 1 Introduction

Parkinson’s disease (PD) is the second most prevalent neurodegenerative disorder worldwide, clinically manifested by bradykinesia, resting tremor, muscular rigidity and postural instability (Trevisan et al., 2024), with classic pathological features including progressive loss of dopaminergic neurons in the substantia nigra pars compacta and intracellular Lewy body formation composed mainly of misfolded and aggregated α-synuclein (α-syn) (Martirosyan et al., 2024). For over a century, PD research has been dominated by a neuron-centric paradigm (Dionísio et al., 2021), with investigations focused on intraneuronal pathological mechanisms, while cerebrovascular pathology was long regarded as a secondary or end-stage consequence of neuronal degeneration, receiving insufficient research attention (Lau et al., 2024; Zhu B. et al., 2024).

In recent years, the emergence and development of the neurovascular unit (NVU) concept have reshaped the understanding of PD pathophysiology (Lau et al., 2024), shifting research focus from isolated neuronal damage to the integrated dysfunction of the NVU—a complex multicellular structure centered on the blood-brain barrier (BBB). Post-mortem and in vivo imaging studies have consistently identified significant cerebrovascular abnormalities in PD patients, including brain microvascular endothelial cell (BMEC) degeneration, basement membrane thickening and reduced capillary density (Lamptey et al., 2022). More importantly, animal model studies have confirmed that increased BBB permeability and pericyte activation can precede detectable dopaminergic neuron degeneration, indicating that BBB dysfunction is not merely a passive byproduct of PD, but an early pathological event that may initiate and drive disease progression (Lau et al., 2024; Yokoya et al., 2025). BBB disruption enables the infiltration of peripheral neurotoxic substances into the central nervous system (CNS), exacerbating neuroinflammation and neuronal damage, thus forming a vicious cycle that accelerates PD pathological progression (Cohen et al., 2024; Erdõ et al., 2017; Kempuraj et al., 2024). Clarifying the molecular mechanisms underlying BBB dysfunction in PD is therefore crucial for a comprehensive understanding of PD pathogenesis and the development of early therapeutic strategies.

AMP-activated protein kinase (AMPK), a highly conserved serine/threonine kinase in eukaryotes, acts as a cellular “metabolic sensor” that responds to energy stress (e.g., hypoxia, oxidative stress, energy depletion) by regulating anabolic and catabolic processes to maintain cellular energy homeostasis (Qu et al., 2025). Beyond its classical metabolic regulatory functions, AMPK exerts complex and multifaceted effects on vascular endothelial cells (Ding et al., 2021; Dragoni et al., 2021): it modulates nitric oxide synthase (eNOS) to promote vasodilation, and regulates cytoskeletal rearrangement and intercellular junction stability to influence endothelial barrier function, which has been well characterized in cardiovascular and metabolic diseases. Notably, α-syn aggregates, a core pathological factor of PD, can induce oxidative stress, endoplasmic reticulum stress and mitochondrial dysfunction—all of which are classic upstream triggers of AMPK activation (Nikbakhtzadeh et al., 2021). This raises the key scientific hypothesis that AMPK may serve as a pivotal signaling molecule linking α-syn cytotoxicity to BBB damage in PD (Ma et al., 2025). However, the specific regulatory mechanisms of AMPK in PD-related NVU injury, including its downstream signaling pathways and substrate proteins in BMECs, remain largely uncharacterized (Manwani and McCullough, 2013). Additionally, AMPK signaling exhibits a context-dependent “double-edged sword” nature, with protective or detrimental effects likely dependent on disease stage, cell type and activation duration—an issue that has not been systematically addressed in the context of chronic neurodegeneration such as PD (Turkseven and Ertuna, 2013).

Unlike previous reviews that focus on AMPK’s metabolic roles in the CNS (Wong et al., 2022; Zhang et al., 2023), this article systematically positions AMPK as a multifunctional signaling hub in PD-associated neurovascular injury. We first summarize the pathological evidence of early BBB injury in PD and the direct toxic mechanisms of α-syn on BMECs; then discuss the known regulatory effects of AMPK on endothelial barrier function and its potential dual role in PD; further highlight the application value of advanced phosphoproteomic techniques in unbiasedly identifying AMPK’s downstream phosphorylation signaling networks in PD models; and finally propose strategies to distinguish the specific effects of AMPK from general stress responses, as well as future research directions for verifying the AMPK-mediated signaling axis in PD-related BBB dysfunction. This review aims to provide a novel “metabolism-signaling” integrated perspective for understanding PD neurovascular pathology, and to lay a theoretical foundation for identifying potential novel targets for early PD intervention by targeting AMPK-regulated NVU protection.

## 2 Paradigm shift in PD research: from neuron-centric to neurovascular unit-oriented perspective

### 2.1 The traditional neuron-centric paradigm: core focus and inherent limitations

For over a century, PD research has been dominated by a neuron-centric paradigm, emphasizing canonical neuropathological hallmarks such as the misfolding and aggregation of α-syn, progressive degeneration of dopaminergic neurons in the substantia nigra pars compacta, and the formation of Lewy bodies composed of aggregated α-syn (Mehra et al., 2019). This framework has profoundly shaped diagnostic criteria and therapeutic strategies, yet it has largely marginalized non-neuronal contributions to disease pathogenesis. Notably, cerebrovascular and neurovascular alterations have historically been interpreted as secondary consequences of neuronal loss rather than potential drivers of early pathology (Koeglsperger et al., 2023), thereby limiting comprehensive mechanistic inquiry into the multifactorial origins of PD.

Emerging evidence now challenges this reductionist view, revealing that pathological processes in PD extend beyond neuronal compartments. Single-nucleus transcriptomic and proteomic analyses of postmortem PD brains have uncovered significant immune activation, including elevated brain-resident T cells and disrupted neuron-astrocyte interactions, alongside widespread synaptic protein downregulation in the prefrontal cortex (Zhu B. et al., 2024). These findings underscore that neuroinflammation and glial dysfunction are integral components of PD pathology, occurring concurrently with or even preceding overt neuronal degeneration. Moreover, α-syn pathology has been shown to inversely correlate with chaperone expression in excitatory neurons, suggesting that proteostatic failure may be an early event in selectively vulnerable circuits (Zhu B. et al., 2024).

Critically, the temporal sequence of pathological events appears more complex than previously assumed. Studies of incidental Lewy body disease—a presumed prodromal stage—demonstrate that molecular and cellular abnormalities, including mitochondrial dysfunction and lysosomal impairment, manifest prior to Lewy body formation and substantial neuronal loss (Koeglsperger et al., 2023). Additionally, experimental models support the Braak hypothesis, wherein pathologic α-syn propagates from the gut to the brain via the vagus nerve, inducing neurodegeneration in a spatiotemporally defined pattern that begins outside the substantia nigra (Bai et al., 2025). This transneuronal spread mechanism implies that PD initiation may originate in peripheral tissues, further undermining the notion that neuronal damage is the sole primary event.

The recognition of early synaptic dysfunction and inflammation, which precede α-syn deposition and aggregation, has shifted research focus toward pre-degenerative mechanisms (Calabresi et al., 2023). Inflammatory cascades, potentially triggered by genetic-environmental interactions, may disrupt calcium homeostasis and mitochondrial bioenergetics, creating a permissive environment for α-syn misfolding (Calabresi et al., 2023). Such insights highlight that PD pathogenesis involves a dynamic interplay between proteinopathy, cellular stress responses, and non-cell-autonomous signaling, rather than a linear cascade originating exclusively within neurons.

Consequently, the traditional neuron-centric model is increasingly viewed as insufficient for capturing the full spectrum of PD etiology. A more integrative framework—one that incorporates vascular, immune, and systemic factors alongside neuronal vulnerability—is essential to identify true initiating events and develop disease-modifying interventions. Recent advances in α-syn seed amplification assays and multimodal imaging now enable biological definition and staging of PD prior to clinical symptom onset (Picca et al., 2021), offering unprecedented opportunities to interrogate early pathophysiological networks that extend well beyond the neuron.

### 2.2 Paradigm update: neurovascular unit concept and evidences for early blood-brain barrier injury in PD

The conceptualization of the neurovascular unit (NVU) has fundamentally reshaped the investigation of central nervous system disorders, positioning blood-brain barrier (BBB) dysfunction as a pivotal pathophysiological element in Parkinson’s disease (PD) rather than a mere epiphenomenon (Lau et al., 2024). Early post-mortem analyses have consistently revealed structural vascular abnormalities in PD brains, including endothelial degeneration, basement membrane thickening, and elevated string vessel formation—collapsed capillary remnants lacking endothelium—particularly within the substantia nigra (Yang et al., 2015). These morphological alterations signify profound microvascular compromise and are closely associated with astrocytic activation and fibrinogen extravasation, indicative of BBB leakage (Yang et al., 2015).

Recent mechanistic insights from human-based models further substantiate the causal role of peripheral factors in BBB disruption. Erythrocyte-derived extracellular vesicles (EEVs) from PD patients have been shown to transcytose across the human BBB via a caveolin-dependent pathway, directly impairing tight junction integrity by downregulating ZO-1 and claudin-5 in brain microvascular endothelial cells (BMECs) (Denis et al., 2025). Critically, the severity of clinical symptoms correlates with both the extent of barrier breakdown and the degree of EEV-mediated dopaminergic neuron atrophy, suggesting a dynamic interplay between systemic pathology and central vulnerability (Denis et al., 2025).

Animal and cellular studies corroborate that α-synuclein pathology actively drives BBB impairment. Accumulation of misfolded α-synuclein disrupts endothelial transporter and tight junction protein expression, triggering a self-perpetuating cycle of inflammation and vascular degeneration that exacerbates neurodegeneration (Lau et al., 2024). This cascade is amplified by activated pericytes and glial cells within the NVU, which release pro-inflammatory mediators that further destabilize barrier function (Kempuraj et al., 2024). Notably, such BBB alterations may precede significant neuronal loss, positioning vascular dysfunction as an early driver rather than a late consequence of PD progression (Denis et al., 2025; Lau et al., 2024).

Therapeutic strategies now increasingly target BBB modulation. Low-intensity focused ultrasound (LIFU), particularly when combined with microbubbles, has demonstrated the capacity to transiently and reversibly open the BBB, facilitating targeted drug delivery while simultaneously exerting neuromodulatory effects through anti-inflammatory and neurotrophic mechanisms (Zhong et al., 2023). A recent phase I trial in Parkinson’s disease dementia confirmed the safety and feasibility of MR-guided focused ultrasound–induced BBB opening in cortical regions, with preliminary evidence of cognitive stabilization (Gasca-Salas et al., 2021). These approaches underscore the BBB not only as a pathological gateway but also as a therapeutic conduit.

Collectively, converging evidence from neuropathology, human cell models, and preclinical interventions redefines BBB dysfunction in PD as an early, active, and potentially modifiable component of disease pathogenesis (Kempuraj et al., 2024; Lau et al., 2024). The integration of NVU-focused biomarkers, including GFAP, PDGFRβ, and neurofilament light, may enable earlier detection of vascular compromise, whereas region-specific BBB alterations could support the precise targeting of vulnerable neuronal populations (Kempuraj et al., 2024). Future therapeutics must therefore account for the dual role of the BBB: as a barrier to drug delivery and as a dynamic participant in the neurodegenerative cascade.

### 2.3 Emerging research hypothesis: AMPK as a central signaling hub linking energy metabolism and neurovascular injury

Concurrently, the pivotal role of energy metabolic dysregulation in the pathogenesis of neurodegenerative diseases has garnered escalating scientific attention. As a master regulator of cellular energy homeostasis, the AMP-activated protein kinase (AMPK) has well-established its regulatory functions in vascular endothelial biology across cardiovascular and metabolic disorders (Chen T. et al., 2024; Dennhardt et al., 2019; Fang et al., 2023). In recent years, a growing body of evidence has extended this line of inquiry to implicate AMPK in BBB injury within various neurological disorders, notably including Alzheimer’s disease.

Given the well-documented capacity of α-synuclein (α-syn) aggregates to elicit oxidative stress and endoplasmic reticulum (ER) stress—both of which are canonical triggers for AMPK activation—and considering AMPK’s well-characterized role in modulating cellular tight junctions and cytoskeletal architecture, a compelling mechanistic hypothesis has emerged: AMPK may function as a critical signaling hub that transduces α-syn cytotoxicity into early BBB compromise in PD (Zhang et al., 2024; Zhu J. et al., 2024). Currently, direct experimental validation of this core hypothesis remains in its formative stages, thereby representing a cutting-edge and rapidly evolving frontier in contemporary PD research (Chen X. et al., 2024; Sun et al., 2023).

**FIGURE 1:**
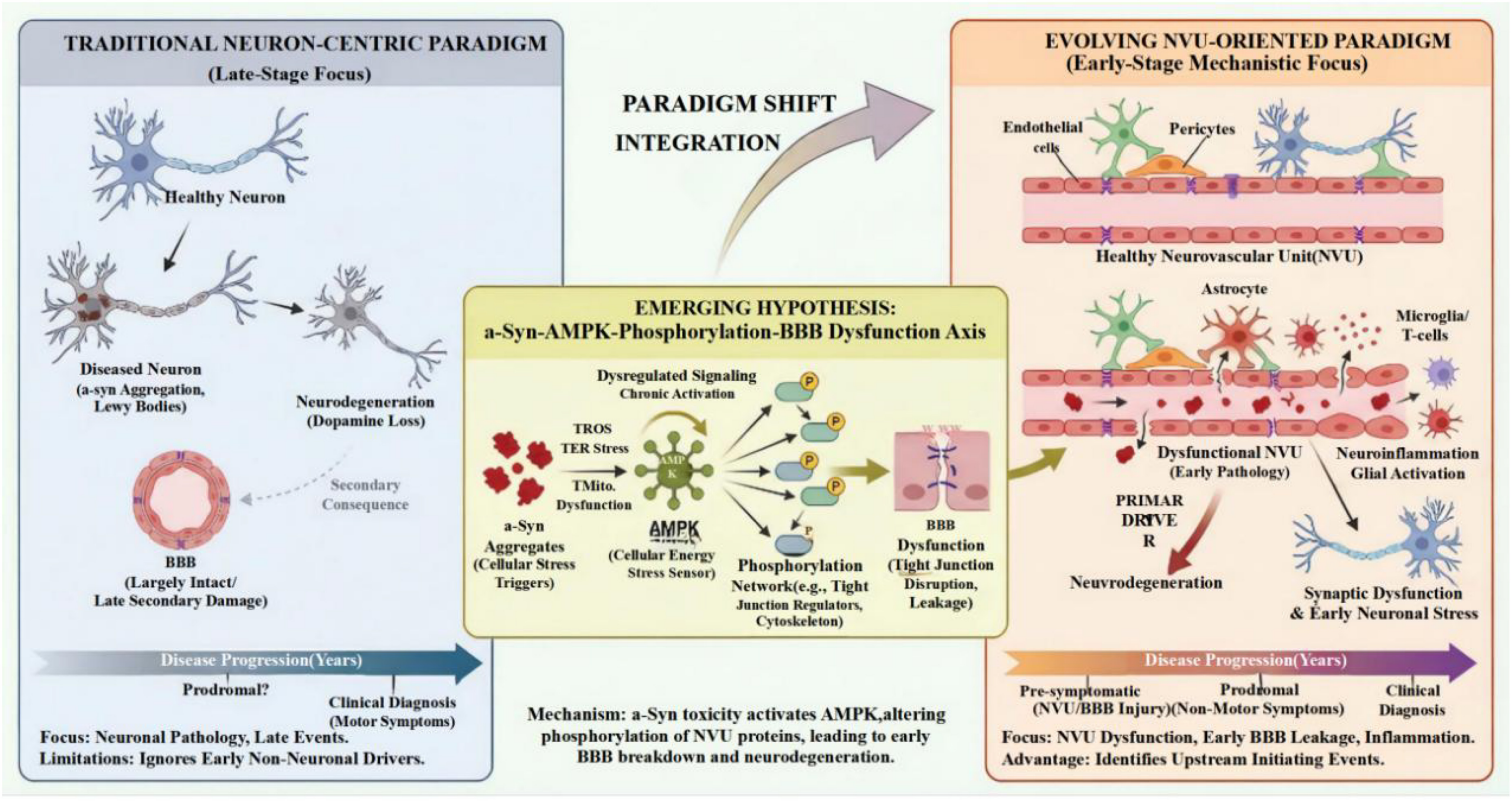
Paradigm shift in Parkinson’s disease (PD) research: from neuron-centric to neurovascular unit-oriented perspective. **(A)** The traditional neuron-centric paradigm focuses on late-stage neuronal pathology (α-synuclein aggregation, Lewy body formation) and treats blood-brain barrier (BBB) changes as secondary events. **(B)** The evolving neurovascular unit (NVU)-oriented paradigm recognizes early BBB injury as an active driver of pathogenesis, involving multiple cell types including endothelial cells, pericytes, astrocytes, and microglia. **(C)** The emerging hypothesis proposes that α-synuclein aggregates trigger cellular stress (oxidative stress, ER stress, mitochondrial dysfunction), activating AMP-activated protein kinase (AMPK) as a central signaling hub. This leads to altered phosphorylation of NVU proteins and subsequent early BBB breakdown, which precedes clinical diagnosis. The bottom timeline illustrates the shift in focus from late-stage neuronal pathology to pre-symptomatic NVU/BBB injury as upstream initiating events in PD pathogenesis.

With the emerging significance of early BBB injury in PD pathogenesis established and the central role of AMPK proposed, the subsequent section will delve into the current state of knowledge regarding the molecular mechanisms underpinning this neurovascular dysfunction. The conceptual evolution from a traditional neuron-centric framework to an integrated neurovascular unit perspective, along with the emerging hypothesis positioning AMPK as a central signaling hub linking α-synuclein toxicity to early BBB dysfunction, is schematically summarized in Figure 1. This illustration highlights the temporal sequence of pathological events and underscores the potential of targeting NVU components in the pre-symptomatic phase of Parkinson’s disease.

## 3 Current research progress: from phenotypic observation to mechanistic exploration of aMPK-mediated BBB dysfunction in PD

### 3.1 Technological innovation-driven dynamic assessment of BBB function in PD progression

Emerging evidence underscores the critical role of blood-brain barrier (BBB) dysfunction in the pathogenesis and progression of PD, with dynamic alterations in BBB integrity now recognized as a key contributor to neurodegeneration (Lau et al., 2024). Preclinical models, particularly transgenic mice overexpressing α-synuclein such as the A53T strain, have demonstrated that oligomeric α-synuclein accumulation in astrocytes triggers vascular endothelial growth factor A (VEGFA) release, leading to tight junction disruption and increased BBB permeability—mechanisms corroborated in postmortem PD brain tissue (Lan et al., 2022). These findings highlight the utility of α-synuclein-based rodent models in dissecting the molecular cascade linking proteinopathy to neurovascular compromise, thereby providing a platform for mechanistic and therapeutic exploration (Lau et al., 2024).

Concurrently, *in vivo* imaging modalities—including dynamic contrast-enhanced magnetic resonance imaging (DCE-MRI) and translocator protein positron emission tomography (TSPO-PET)—are advancing the non-invasive assessment of BBB permeability and neuroinflammation across PD stages (Kovbasyuk et al., 2025). While these techniques offer spatial and temporal resolution of BBB dynamics in humans, methodological heterogeneity in tracer selection, input function modeling, and parameter estimation currently limits cross-study comparability and clinical translation (Kovbasyuk et al., 2025). Nevertheless, such approaches hold promise for longitudinal monitoring of BBB integrity and may serve as surrogate endpoints in trials targeting neurovascular pathways.

Clinically, cerebrospinal fluid (CSF) biomarkers provide compelling evidence of angiogenic dysregulation and BBB damage in PD. Some studies have reported elevated levels of angiogenic factors, such as vascular endothelial growth factor (VEGF) and placental growth factor (PIGF), in the cerebrospinal fluid of PD patients, suggesting the activation of vascular leakage or remodeling processes (Al-Bachari et al., 2020; Janelidze et al., 2015; Sweeney et al., 2019). A cross-sectional study within the Swedish BioFinder cohort (*n* = 100 PD patients, *n* = 38 controls) revealed significantly elevated CSF levels of VEGF and PlGF in both PD patients with and without dementia, compared to age-matched controls (Janelidze et al., 2015).

Collectively, convergent data from animal models, neuroimaging, and human biofluid studies implicate aberrant angiogenic signaling—particularly via the VEGF/VEGFR axis—as a central mechanism underlying BBB disruption in PD (Al-Bachari et al., 2020; Nation et al., 2019; Sweeney et al., 2019). While VEGF exerts neurotrophic effects at physiological levels, its pathological overexpression, driven by α-synuclein-activated astrocytes, appears to promote vascular leakage and neuroinflammation (Sweeney et al., 2019). This dual role necessitates precision therapeutic strategies that modulate VEGF signaling without compromising its neuroprotective functions—a challenge that underscores the need for biomarker-guided interventions and region-specific BBB-targeted delivery systems (Al-Bachari et al., 2020).

### 3.2 Elucidation of α-synuclein-mediated toxic mechanisms in brain microvascular endothelial cells

#### 3.2.1 α-synuclein-induced cellular stress: a trigger for AMPK activation in neurovascular unit

Research has advanced beyond the mere observation of tight junction protein downregulation to unravel the diverse molecular pathways by which α-synuclein aggregates exert cytotoxic effects on brain microvascular endothelial cells. These aggregates can disrupt intracellular vesicle trafficking, impeding the proper localization of tight junction proteins to the cell membrane. Concurrently, they can induce mitochondrial dysfunction, resulting in excessive production of reactive oxygen species (ROS) and triggering oxidative stress. Furthermore, α-syn aggregates can cause comprehensive impairment of endothelial cell function by triggering endoplasmic reticulum (ER) stress and activating its downstream signaling cascades (Bogale et al., 2021; Hourfar et al., 2023; Kuan et al., 2016).

#### 3.2.2 Context-dependent dual role of AMPK: a double-edged sword in PD-related BBB regulation

The accumulation of α-synuclein aggregates in the neurovascular unit triggers multiple forms of cellular stress, including oxidative stress, ER stress, and mitochondrial dysfunction. These stressors are known to activate AMPK through upstream kinases such as LKB1 and CaMKKβ, primarily in response to an increased AMP/ATP ratio and elevated cytosolic Ca^2 +^ levels (Hardie et al., 2012; Herzig and Shaw, 2018). In BMECs, AMPK activation exhibits a dual role in BBB homeostasis: it may initially serve as a protective adaptive response to metabolic stress by enhancing tight junction integrity and suppressing oxidative damage (Ma et al., 2025). However, under chronic pathological conditions, sustained AMPK signaling could paradoxically contribute to cytoskeletal destabilization and junctional disassembly. This biphasic effect is supported by evidence showing that AMPK activation reinforces BBB integrity in models of acute insult (Ma et al., 2025); yet, the long-term consequences of persistent AMPK stimulation in neurodegenerative contexts—particularly in PD, where α-synuclein pathology drives chronic vascular stress—remain poorly characterized.

Elucidating how α-synuclein–mediated proteotoxic stress interfaces with AMPK-dependent signaling pathways is therefore critical to understanding cerebrovascular dysfunction in PD. Although current literature on AMPK in vascular contexts is limited in PD, emerging insights suggest that dysregulated AMPK signaling may constitute a convergent node linking proteinopathy, bioenergetic failure, and vascular leakage. Given that traumatic brain injury (TBI)-induced BBB disruption increases the risk of PD and other synucleinopathies, and that AMPK-targeted interventions like P7C3-A20 restore endothelial continuity months after injury (Vázquez-Rosa et al., 2020), it is plausible that the temporal dynamics of AMPK activation determine whether this pathway acts as a therapeutic target or a maladaptive liability. Future studies must therefore delineate the context-dependent outcomes of AMPK activation in α-synuclein–exposed BMECs to inform targeted vascular protection strategies in PD.

AMP-activated protein kinase (AMPK) has demonstrated barrier-protective effects by regulating pathways such as caveolin-1 (Cav-1) and matrix metalloproteinase-9 (MMP-9) in acute injury models, such as cerebral ischemia (Dennhardt et al., 2019; Marinangeli et al., 2016). However, the majority of these studies concentrate on acute injury models, with limited research conducted within the context of chronic neurodegenerative conditions like PD. Although AMPK activation has primarily been associated with barrier-protective effects in acute injury models, emerging evidence suggests that its role in chronic neurodegenerative conditions such as PD may be context-dependent. Prolonged or dysregulated AMPK activation could contribute to endothelial dysfunction, autophagic dysregulation, or even cell death, thereby exacerbating BBB breakdown (Liu et al., 2018; Sun et al., 2023). The dual nature of AMPK signaling—exerting both neuroprotective and potentially detrimental effects depending on cellular context and disease stage—represents a critical yet underexplored dimension in PD pathogenesis, particularly within the neurovascular unit (Lau et al., 2024). While AMPK activation has been shown to enhance autophagy-mediated clearance of α-synuclein aggregates and mitigate oxidative stress in dopaminergic neurons (Lau et al., 2024), emerging evidence suggests that chronic or dysregulated AMPK activity may impair mitochondrial biogenesis and exacerbate energy deficits in vulnerable neuronal populations. This functional duality underscores the necessity for temporally resolved and cell-type-specific interrogation of AMPK dynamics, as global modulation could inadvertently disrupt homeostatic processes essential for neuronal survival.

### 3.3 Pharmacological cues from traditional Chinese medicine and the urgency of PD-specific mechanistic validation

Domestic research in this field primarily concentrates on the construction of PD animal models and the validation of the pharmacological effects of active ingredients in traditional Chinese medicine (TCM) (Begh et al., 2025). For instance, berberine, a well-known AMPK activator, has demonstrated efficacy in improving motor deficits and reducing BBB leakage in PD models, accompanied by alterations in AMPK pathway phosphorylation levels (Wang et al., 2026). Other investigations have explored the protective potential of TCM compounds on the neurovascular unit (NVU) by modulating AMPK-related pathways (Nematalla et al., 2025). While these pharmacological observations suggest AMPK involvement, they do not yet establish a direct mechanistic link from α-syn toxicity through AMPK to specific phosphorylation events. To rigorously validate this signaling axis, future studies should employ PD-specific experimental models—such as BMECs treated with α-syn PFFs or Thy1-αSyn transgenic mice—combined with AMPK-specific knockout or pharmacological interventions. Such approaches will enable the systematic assessment of BBB permeability and the characterization of the AMPK-dependent phosphoproteome in PD-related neurovascular injury (Elabi et al., 2021; Kuan et al., 2016).

## 4 Key scientific gaps and integrated research prospects

### 4.1 Mechanistic ambiguities and the unique value of phosphoproteomics in deciphering AMPK downstream signaling

The mechanistic role of AMPK in BBB dysfunction associated with neurodegenerative pathologies remains incompletely defined, particularly regarding whether it functions as a passive signaling relay or an active executor in the cascade linking pathological triggers to tight junction destabilization. While emerging evidence implicates AMPK activation in preserving BBB integrity under diverse stressors—including amyloid-β toxicity, lipopolysaccharide (LPS)-induced inflammation, and hyperglycemia—its precise position within the signaling hierarchy and its full repertoire of phosphorylation substrates in brain microvascular endothelial cells (BMECs) are still unclear (Cai et al., 2025; Cui et al., 2024; Ma et al., 2025). Notably, multiple studies demonstrate that AMPK activation upregulates key tight junction proteins such as occludin, claudin-5, and ZO-1, thereby attenuating paracellular permeability (Hu and Liu, 2016; You et al., 2019; Yu et al., 2015). However, these findings do not resolve whether AMPK acts upstream as a modulator or downstream as a direct effector of cytoskeletal and junctional remodeling. The key to addressing these questions lies in the application of innovative methodologies. Future research should prioritize the use of conditional gene knockout mice and specific inhibitors/activators to ascertain the necessity and direction of AMPK’s role in PD-BBB injury *in vivo*. The pivotal breakthrough point is the application of phosphoproteomics technology. By comparing brain microvascular endothelial cells (BMECs) with varying states of AMPK activity under PD model conditions, researchers can unbiasedly identify AMPK-dependent differential phosphorylation sites, thereby constructing and validating its downstream signaling network (Chen H. et al., 2025; Li et al., 2021; Veys et al., 2020; Wu et al., 2024).

To delineate AMPK-specific phosphorylation events from the confounding signaling alterations induced by oxidative stress in PD models, a multi-tiered experimental and computational strategy is essential. Pharmacological modulation of AMPK activity using selective inhibitors such as BAY-3827—which stabilizes an inactive kinase conformation through a unique disulfide bridge mechanism—and activators like AICAR enables causal inference of AMPK-dependent signaling (Bringas et al., 2025). Complementing this, genetic ablation of AMPKα1/α2 subunits in cellular models provides a definitive platform to isolate the AMPK-regulated phosphoproteome. This approach, when integrated with in silico motif screening based on the canonical AMPK consensus sequence (ϕ-X-β-S/T-X-X-ϕ, where ϕ is hydrophobic and β is basic), significantly enhances the specificity of substrate identification (Marin et al., 2015). Furthermore, the research perspective should expand from BMECs to include other NVU cell types like pericytes and astrocytes, considering their role in intercellular communication and metabolic reprogramming (Han et al., 2025; Oikari et al., 2020; Zhao et al., 2020).

### 4.2 Technical challenges in phosphoproteomic research and its clinical translational significance

Phosphoproteomics represents a powerful, unbiased methodology for delineating AMPK-dependent signaling networks with high resolution; however, its application in complex neurological contexts is constrained by several technical hurdles. Chief among these are the necessity for cell-type-specific sampling within the heterogeneous NVU, the transient nature of phosphorylation events that demands precise temporal resolution, and the substantial computational burden associated with data interpretation. Recent advances in microfluidic-integrated phosphoproteomic platforms, such as Chip-DIA, have demonstrated unprecedented sensitivity in mapping nanoscale-to-single-cell phosphoproteomes, enabling the detection of over 10,000 phosphopeptides from fewer than 1,000 cells and revealing druggable signaling nodes in cancer (Muneer et al., 2025). Nevertheless, adaptation of such ultrasensitive technologies to neurological tissues—particularly for isolating distinct NVU components like endothelial cells, pericytes, astrocytes, and microglia—remains technically demanding and requires specialized protocols such as EPAM-ia, which enables syngeneic isolation of these cell types for downstream omics analyses (Spitzer et al., 2023).

The dynamic and context-dependent nature of phosphorylation further complicates the distinction between functionally consequential signaling events and incidental modifications. To address this, integration of phosphoproteomic datasets with orthogonal functional validation in physiologically relevant *in vivo* models is essential. This integrative approach not only enhances biological interpretability but also facilitates the identification of true effector molecules within dysregulated pathways. For instance, phosphoproteomic profiling of peripheral blood mononuclear cells (PBMCs) across PD stages has successfully uncovered stage-specific molecular signatures, linking phospho-dynamics to clinical progression and underscoring the value of longitudinal, clinically annotated cohorts (Massacci et al., 2024). Similarly, machine learning–driven analyses of phospho-proteomes have yielded highly accurate classifiers (e.g., 96% accuracy) for LRRK2 G2019S carriers, demonstrating the translational potential of phospho-signatures when coupled with robust bioinformatic frameworks (Cortés et al., 2025).

The identification of phosphorylated proteins or sites as biomarkers for early BBB injury holds significant clinical promise. Phosphorylated isoforms of proteins such as RAB12 (pSer106) have already been validated as endogenous readouts of LRRK2 kinase activity in PBMCs from G2019S carriers, with levels correlating with motor severity and responding to pharmacological inhibition (Cortés et al., 2025). Moreover, urinary extracellular vesicles (EVs) have yielded panels of phosphoproteins linked to autophagy, neuroinflammation, and amyloid fibril formation—key pathways in PD—highlighting the feasibility of non-invasive phospho-biomarker discovery (Le et al., 2026). These findings align with broader observations that phospho-signatures in accessible biofluids reflect central pathological processes, reinforcing their utility in early diagnosis and therapeutic monitoring.

### 4.3 Theoretical integration and translational potential of the α-syn-AMPK-phosphorylation network-BBB dysfunction axis

Through the aforementioned research, we expect to establish a new pathological axis in PD: “α-syn - AMPK - phosphorylation network - BBB dysfunction” (Chen H. et al., 2025; Li et al., 2021; Motzer et al., 2020). This axis not only fills the theoretical gap in the understanding of how PD core pathological factors induce early neurovascular injury, but also redefines the positional role of AMPK from a simple cellular metabolic sensor to a key signaling hub (Kim et al., 2025) that bridges proteotoxic stress and vascular barrier dysfunction in PD. The establishment of this axis deepens the cognitive understanding of early PD pathological events, breaking the traditional research bottleneck that separates neuronal proteinopathy from neurovascular damage, and providing a more integrated and systematic theoretical framework for interpreting the multi-component and multi-pathway pathological characteristics of PD (Kim et al., 2025; Li N. et al., 2025; Liu et al., 2025; Ni et al., 2025; Xie et al., 2023).

At the clinical translational level, the α-syn-AMPK-phosphorylation network-BBB dysfunction axis has important application value in the development of early PD diagnosis biomarkers and targeted therapeutic strategies (Chen S. et al., 2025; Zhang et al., 2025). On the one hand, the key phosphorylated proteins or specific phosphorylation sites of the AMPK downstream signaling network identified by phosphoproteomics can serve as novel molecular biomarkers for the diagnosis of early PD BBB injury (Chen S. et al., 2025). Unlike the currently used clinical symptoms and conventional imaging markers, these phosphorylation-specific biomarkers can reflect the early neurovascular pathological changes of PD before the occurrence of obvious dopaminergic neuron loss, which is conducive to the early screening and staging diagnosis of PD, and provides a reliable biological basis for the implementation of early intervention. On the other hand, this axis provides precise and multiple potential therapeutic targets for PD neurovascular protection (Chen Z. X. et al., 2025; Hou et al., 2021; Kang et al., 2017).

Traditional PD therapeutic strategies mostly focus on improving dopaminergic neurotransmission and inhibiting α-syn aggregation, while ignoring the regulation of neurovascular injury. Based on this new axis, we can develop targeted therapeutic strategies that regulate the AMPK phosphorylation network: for example, designing small molecule drugs that precisely activate or inhibit specific downstream effectors of AMPK (Kang et al., 2017; Pérez-Revuelta et al., 2014), so as to enhance the protective effect of AMPK on the BBB while avoiding the adverse effects of global AMPK modulation on cellular metabolic homeostasis; or screening traditional Chinese medicine active ingredients that can specifically regulate the AMPK phosphorylation pathway, combining the advantages of multi-component and multi-target regulation of traditional Chinese medicine to achieve the comprehensive protection of the NVU. In addition, the combination of AMPK pathway-targeted drugs with BBB opening technologies (such as low-intensity focused ultrasound) can further improve the targeted delivery efficiency of drugs to the brain tissue, and enhance the therapeutic effect of PD by simultaneously regulating the AMPK phosphorylation network and repairing the damaged BBB structure.

The multifaceted intervention strategies targeting the AMPK signaling axis, ranging from pharmacological modulators and natural compounds to physical techniques and biomarker development, are systematically summarized in Table 1, highlighting their mechanisms, effects on BBB function, and translational potential in PD.

**TABLE 1:** Therapeutic and diagnostic strategies targeting the AMP-activated protein kinase (AMPK) signaling axis in Parkinson’s disease (PD)-associated neurovascular injury.

Intervention category	Specific agent/modality	AMPK modulation	Molecular target/substrate	Effect on BBB function	Translational potential	References
Western pharmaceutical	Metformin	Activation	AMPK → PP2A activation	Reduces p-Ser129 α-synuclein levels; indirect BBB protection via reduced proteinopathy	High (clinically approved for diabetes; repurposing potential)	Pérez-Revuelta et al., 2014
Western pharmaceutical	AICAR	Direct activation	AMPK (direct activator)	Upregulates tight junction proteins (ZO-1, claudin-5); reduces paracellular permeability	Preclinical (limited by bioavailability)	Bringas et al., 2025
Western pharmaceutical	BAY-3827	Selective inhibition	AMPK (disulfide bridge-mediated inactivation)	Research tool to dissect AMPK-dependent vs. independent effects; no direct therapeutic use yet	Low (primarily experimental)	Bringas et al., 2025
Traditional Chinese medicine	Berberine	Activation	AMPK pathway (upstream)	Reduces BBB leakage; improves motor deficits in PD models	High (extensive safety profile; clinical trials ongoing)	Nematalla et al., 2025; Wang et al., 2026
Traditional Chinese medicine	Ginsenoside Rh4 (from *Panax ginseng*)	Activation	AMPK-eNOS signaling	Endothelial protection; promotes vasodilation; maintains barrier integrity	Moderate (requires formulation optimization)	Zhang et al., 2024
Traditional Chinese medicine	Total flavonoids (from *Prunus mume*)	Activation	CaMKKβ/AMPK pathway	Promotes neuronal mitophagy; indirect NVU protection	Moderate (preclinical evidence)	Wang et al., 2026
Traditional Chinese medicine	Stigmasterol	Activation	AMPK → NF-κB/NLRP3 inhibition	Attenuates microglial inflammation; stabilizes NVU	Low (early preclinical)	Jie et al., 2022
Physical/device-based	LIFU + microbubbles	Indirect (cellular stress response)	BBB opening (reversible tight junction modulation)	Transient BBB opening for drug delivery; may trigger protective AMPK signaling	High (Phase I trials completed in PDD)	Gasca-Salas et al., 2021; Zhong et al., 2023
Physical/device-based	Photobiomodulation	Activation	AMPK pathway	Mitigates BBB disruption in AD models; potential applicability to PD	Moderate (early clinical for other indications)	Ma et al., 2025
Biomarker	p-RAB12 (Ser106)	Readout of LRRK2 activity (AMPK crosstalk)	RAB12 phosphorylation	Detected in PBMCs; correlates with motor severity; reflects kinase dysregulation	High (validated in G2019S carriers)	Cortés et al., 2025
Biomarker	Phosphoproteomic signatures	Network-level analysis	Multiple AMPK substrates (e.g., tight junction proteins, cytoskeletal regulators)	Identifies early BBB injury markers; enables staging of vascular pathology	Moderate (requires validation in large cohorts)	Le et al., 2026; Massacci et al., 2024; Muneer et al., 2025; Spitzer et al., 2023
Biomarker	Urinary EV phosphoproteins	Indirect (autophagy/ neuroinflammation pathways)	Phosphoproteins linked to autophagy and amyloid fibril formation	Non-invasive detection of PD-related pathways	Moderate (emerging technology)	Le et al., 2026

## 5 Summary and outlook

In summary, this review systematically elucidates the pivotal role of AMPK in neurovascular unit injury associated with PD, shifting the research perspective from the traditional neuron-centric paradigm to an integrated neurovascular focus and positioning BBB dysfunction as an early and active driver of PD pathogenesis rather than a secondary consequence of neuronal degeneration. We clarify that α-synuclein aggregates, the core pathological factor of PD, induce oxidative stress, endoplasmic reticulum stress and mitochondrial dysfunction in the neurovascular unit, thereby activating AMPK—a master regulator of cellular energy homeostasis that also modulates endothelial tight junction integrity and cytoskeletal dynamics. AMPK exhibits a context-dependent “double-edged sword” role in PD-related BBB regulation: its moderate activation may serve as a compensatory protective mechanism to maintain endothelial barrier function in the early stage of the disease, while sustained or dysregulated activation under chronic pathological stress can trigger aberrant phosphorylation of key proteins, exacerbating BBB disruption and neurovascular injury. Additionally, we highlight the pharmacological clues from traditional Chinese medicine active ingredients targeting the AMPK pathway, and emphasize the unique value of phosphoproteomic technology in deciphering AMPK’s downstream phosphorylation signaling networks, which lays a theoretical foundation for constructing the novel pathological axis of α-syn-AMPK-phosphorylation network-BBB dysfunction in PD. Collectively, these findings reveal AMPK as a multifunctional signaling hub bridging α-syn proteotoxicity and neurovascular injury, and provide a novel “metabolism-signaling” integrated perspective for understanding PD’s early pathophysiological mechanisms.

Despite the significant progress in understanding the association between AMPK and PD-related neurovascular injury, notable research limitations and unresolved scientific questions remain, pointing to clear directions for future investigations. A key limitation is the lack of in-depth characterization of the divergent roles of AMPK isoforms (α1/α2) in different neurovascular unit cell types (endothelial cells, pericytes, astrocytes) and across distinct PD disease stages, with the specific downstream phosphorylation substrates and signaling cascades of AMPK in brain microvascular endothelial cells yet to be fully identified. Moreover, the molecular mechanisms underlying the transition of AMPK from a protective to a pathogenic role under chronic PD stress remain unclear, and the crosstalk between AMPK and other key signaling pathways regulating BBB function has not been systematically elucidated. Future research should first employ cell-type-specific gene knockout models and stage-controlled PD animal models to clarify the stage-specific and cell-type-specific effects of AMPK activation on BBB function, combining pharmacological interventions to define the therapeutic time window for AMPK modulation. Second, leverage high-resolution phosphoproteomic technology combined with bioinformatic analysis to unbiasedly identify AMPK-dependent phosphorylation events and construct its downstream signaling network in PD models, distinguishing AMPK-specific effects from general stress responses. Third, explore the intercellular communication mechanisms mediated by AMPK in the neurovascular unit, including its regulation of pericyte-endothelial cell interaction, exosome secretion and metabolic reprogramming, to fully elucidate the integrated regulatory role of AMPK in neurovascular homeostasis (Jie et al., 2022; Stevanovic et al., 2024; Zhang et al., 2021). Finally, translate these basic research findings into clinical practice by validating AMPK pathway-related phosphorylated proteins as early biomarkers for PD BBB injury (Li Y. et al., 2025), and developing isoform-specific AMPK modulators or combining TCM active ingredients with BBB-targeted delivery technologies to design novel neurovascular protective therapeutic strategies for PD, which is expected to open up new avenues for early intervention and disease-modifying treatment of PD.

## Data Availability

The original contributions presented in the study are included in the article/supplementary material, further inquiries can be directed to the corresponding author.
